# Dynamic analysis of Th1/Th2 cytokine concentration during antiretroviral therapy of HIV-1/HCV co-infected Patients

**DOI:** 10.1186/1471-2334-12-102

**Published:** 2012-04-25

**Authors:** Wenzhen Kang, Yuan Li, Yan Zhuang, Ke Zhao, Dedong Huang, Yongtao Sun

**Affiliations:** 1 Department of Infectious Diseases, Tangdu Hospital Affiliated to the Fourth Military Medical University, Xi'an, P.R. China

## Abstract

**Background:**

Co-infection with hepatitis C (HCV) is very common in human immunodeficiency virus 1 (HIV-1) infected patients. Although HIV co-infection clearly accelerates progression of HCV-related fibrosis and liver disease, controversy remains as to the impact of HCV on HIV disease progression in co-infected patients. HIV can cause immune dysfunction, in which the regulatory function of T helper (Th) cells is very essential. Moreover, cytokines derived from Th cells play a prominent role in viral infection. Investigating the functional changes of Th1 and Th2 cells in cytokine level can improve the understanding of the effect of co-infected HCV on HIV infection.

**Methods:**

In this study, we measured the baseline Th1/Th2 cytokine concentration in sera by using flow cytometry in HIV/HCV co-infection, HIV mono-infection, HCV mono-infection, and healthy control group, as well as the dynamic changes of these cytokine levels after receiving highly active antiretroviral therapy (HAART).

**Results:**

The ratio of Th1 and Th2 cytokine concentration in HIV/HCV co-infection was higher than HCV mono-infection and healthy control group, while lower than HIV mono-infection group. After HAART was initiated, the Th1/Th2 ratio of HIV/HCV co-infection group decreased to the same level of healthy control, while HIV mono-infection group was still higher than the control group.

**Conclusions:**

There was no significant evidence showing co-infected with HCV had negative effect on HIV related diseases. However, co-infected with HCV can decrease Th1/Th2 ratio by affecting Th1 cytokine level, especially the secretion of IFN-γ. With the initiation of HAART, Th1 and Th2 cytokine levels were progressively reduced. HIV was the main stimulating factor of T cells in HIV/HCV co-infection group.

## Background

Human immunodeficiency virus 1 (HIV-1) co-infected with hepatitis C virus (HCV) is very common because they share the same route of infection. These HIV/HCV co-infected persons account for approximately 25% of all HIV-infected persons all over the world [1]. Injection drug users (IDUs) are shown to be the highest risk factor of HIV/HCV co-infection [2-5]. According to a study investigation performed in 2008, approximately 63.2% of HIV-infected patients were co-infected with HCV in different areas of China [6], and the prevalence was 96.6% in IDUs and 92.9% in former paid blood donors (FBD) [7]. The previous studies indicated that HIV/HCV co-infection was associated with accelerated progression of liver disease and decreased survival rate among HCV-infected individuals comparing with HCV mono-infection [8-10]. Since the widespread and effective introduction of highly active antiretroviral therapy (HAART) has successfully inhibited HIV-related diseases, the chronic liver diseases related to HCV have become one of the major causes of death in HIV/HCV co-infected patients [11,12]. However, studies of the impact of HCV on HIV-infection have opposite conclusions. Some indicated HCV infection has a significant effect on the progression of HIV to AIDS defining illness and AIDS related mortality [13-16], while others found that HCV co-infection has no significant effect on HIV progression [17-22]. Neither of their mechanisms has been defined.

Immunological impairment is the main characteristic of HIV pathogenesis. With the progressive loss of CD4^+ ^T cells in HIV infection, the dysfunction in the T cells compartment is reflected by cytokine expression levels [23-25]. In experimental models, it is widely accepted that susceptibility of BALB/c mice to *L. major *infection is associated with interleukin (IL)-4 and IL-10 produced by Th2 cells, whereas resistance is related to early and persistent interferon (IFN)-γ produced by Th1 cells [26]. Simultaneous production of IFN-γ, tumor necrosis factor (TNF)-α, and IL-10 by antigen-stimulated peripheral blood mononucleaer cells (PBMCs) from patients with active lesions [27] and IL-2, IL-4, IL-5, IL-10, and IFN-γ mRNAs were demonstrated in biopsy samples taken from active lesions [28-30]. IL-10 expression was also significantly higher in patients who responded poorly to pentamidine treatment [28]. Many studies indicated that HIV-induced immunodeficiency often ascribed to a bias of Th1/Th2 balance towards Th2 cytokine responses [31], and this unbalance may recovered slightly when patients received antiretroviral therapy (ART). However, patients with weak immune response before treatment may retain deficiency of immune function, despite of successful inhibition of HIV viral load and increase CD4^+ ^T cell counts, including patients with impaired lymphoproliferative responses, antibody responses to vaccination and cutaneous delayed-type hypersensitivity responses [32]. In addition, HCV-induced liver diseases also affect Th1/Th2 orientation by increasing Th1-type cytokine production [33]. After stimulation by viral peptides or antigen, the Th1 and Th2 cytokine levels were reduced in mono-HIV infected women and more extensively in women with HCV/HIV co-infection when compared with mono-HCV infection [34]. However, the expression profile of Th1/Th2 cytokine in HIV/HCV co-infected patients and their dynamic changes during HAART is rarely known.

In this study, we investigated the cytokine levels putatively produced by Th1 and Th2 cells in HIV/HCV co-infected, mono-HIV and mono-HCV infected patients as the antiviral treatment proceeding. Our prospection is to illustrate the difference of Th1/Th2 unbalance between HIV/HCV co- and mono-infection by correlating the production of cytokines, which would be a convincible evidence of effect of HCV on HIV infected patients.

## Methods

### Study participants

A cohort including four groups in this study was established: HIV/HCV co-infected (n = 20), HCV mono-infected (n = 10), HIV mono-infected (n = 20), and healthy controls (n = 10). For all participants, HCV infection was diagnosed according to the criteria as positive plasma HCV antibodies and detectable baseline HCV RNA (> 50 copies/ml); HIV infection was diagnosed according to the criteria as seropositive for HIV and nadir CD4^+ ^T cell counts with 350 cells/μl. Healthy control subjects were negative for both HCV and HIV antibodies. All study participants were recruited from the Department of Infectious Diseases of Tangdu Hospital (Xi'an, China) and the Guangzhou 8^th ^Hospital (Guangzhou, China). Neither the anti-HIV-positive nor anti-HCV-positive patients had received ART treatment. All studies were conducted with the approval of the ethical review board of each institution. The inform consent was get from each individual in this study.

### Antiretroviral therapy criteria

HIV/HCV co-infected patients started HAART treatment immediately after recruitment if their CD4^+ ^T cell counts were < 200 cells/μl. When the CD4^+ ^T cell count was achieved at > 200 cells/μl and kept stable within 3 months, anti-HCV ART (Peginterferon alfa-2a plus ribavirin) was initiated. If the CD4^+ ^T cell counts were between 200 and 350 cells/μl, HAART was started first, anti-HCV ART was initiated after 12 weeks. HIV and HCV mono-infected patients started HAART and anti-HCV ART treatment directly after recruitment.

### Viral load and lymphocyte subsets test

Both HIV and HCV plasma viral loads were analyzed by the immunofluorescence quantifying PCR assay (Roche Corporation, USA) according to the manufacturer's instructions. T-lymphocyte subsets were enumerated in 50 μl freshly obtained EDTA-anticoagulant whole blood using 20 μl directly labeled mAbs (TriTEST: CD4-FIITC/CD8-PE/CD3-PerCP) (BD Bioscience, USA) according to the "lyse-no-wash" procedure. All samples were acquired with a four-color FACS Calibur and analyzed using MultiSET software (Becton Dickison, USA).

### Th1 and Th2 cytokines test

Six cytokines putatively produced by Th1 and Th2 cells, including IL-2, IL-4, IL-5, IL-10, TNF-α and IFN-γ, were detected using CBA Th1/Th2 cytokine kit (BD™ Cytometric Bead Array, USA) according to the manufacturer's instruction. BD CellQuest software is required for acquiring samples and formatting data. Standard curves and sample cytokine concentration were calculated using the BD CBA Software (BD Bioscience, USA). Th1 cytokine levels were represented by the amount of concentrations of IFN-γ, IL-2 and TNF-α; Th2 cytokine levels were represented by the amount of concentrations of IL-4, IL-5 and IL-10.

### Statistical Analysis

Data were expressed as the Median (interquartile range). Statistical analysis of demographics and laboratory parameters was performed by SPSS 13.0 using standard nonparametric statistical methods (Wilcoxon signed-rank test and Mann-Whitney U test). Statistical analysis of cytokine concentration between groups was performed using GraphPad Prism version 5.00 for Windows (GraphPad Software, San Diego, CA) using the Bonferroni post-test. The differences of Th1/Th2 ratio between groups were compared using the Bonferroni's Multiple Comparison Test. Spearman correlation analysis was performed for correlation analysis. All tests were two-tailed, and *p *values of < 0.05 indicated statistical significance.

## Results

### Study population characteristics and antiretroviral therapeutic situation

Four groups of patients were recruited into this study: HIV/HCV co-infected, HIV mono-infected, HCV mono-infected, and healthy controls. The demographics and laboratory parameters of the four groups are summarized in Table 1. The mean age of HIV/HCV co-infections was older than HIV mono-infections (*p *> 0.05), as well as significantly younger than HCV mono-infections (*p *< 0.05). The main transmission route of HIV/HCV co-infected patients was IDU (80%). The HIV mono-infections has the lowest nadir CD4 T cell count (*p *< 0.05) and highest HIV-1 RNA levels in plasma (*p *< 0.05) comparing with the other three study groups. The HCV RNA levels in plasma of HIV/HCV co-infection group were higher than HCV mono-infection group without statistical difference (*p *> 0.05).

**Table 1:** Baseline demographic and immunological parameters of study participants

Parameters	HIV/HCV co-infection	HIV mono-infection	HCV mono-infection	Healthy control
Number	20	20	10	10
Sex ratio (male/female)	13/7	12/8	7/3	4/6
Age (years)	39.00 (35.25 ~ 41.50) ^c, d^	37.00 (32.75 ~ 41.50) ^c, d^	45.50 (39.50 ~ 48.50) ^a, b, d^	26.50 (25.00 ~ 29.25) ^a, b, c^
Transmission route (no. (%))				
Sex	0 (0)	12 (60)	0 (0)	-
Blood	4 (20)	1 (5)	10 (100)	-
IDU	16 (80)	4 (20)	0 (0)	-
Unknown	0 (0)	3 (15)	0 (0)	-
CD4 T cell count (cells/μl)	224.00 (164.00 ~ 296.00) ^b, c, d^	131.00 (73.75 ~ 194.50) ^a, c, d^	522.50 (448.00 ~ 599.00) ^a, b, d^	817.00 (736.00 ~ 947.25) ^a, b, c^
HIV-1 Viral load (log_10 _copies/ml)	3.97(3.48 ~ 4.83) ^b^	4.65(4.38 ~ 5.23) ^a^	-	-
HCV Viral load (log_10 _copies/ml)	6.42(5.91 ~ 6.85)	-	6.05(5.33 ~ 6.54)	-

a. p < 0.05 difference when compared to HIV/HCV co-infection

b. p < 0.05 difference when compared to HIV mono-infection

c. p < 0.05 difference when compared to HCV mono-infection

d. p < 0.05 difference when compared to healthy control

**Figure 1:**
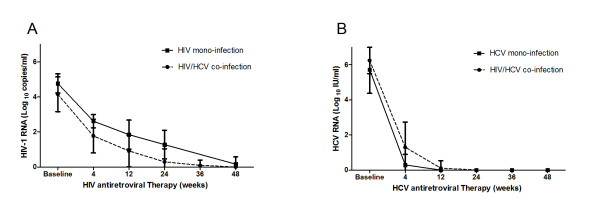
**Changes in plasma viral RNA during antiretroviral therapy between the HIV/HCV co-infection and the HIV or HCV mono-infection group**. Each symbol represents the log_10 _HIV-1 or HCV RNA copies per milliliter of plasma. HIV-1 and HCV RNA levels were measured at six time points: before the start of therapy (baseline) and at 4, 12, 24, 36 and 48 weeks of antiretroviral therapy. (A) Changes in plasma HIV-1 RNA during HAART between the HIV/HCV co-infection (dotted line) and the HIV mono-infection group (solid line). (B) Changes in plasma HCV RNA during HCV-ART between the HIV/HCV co-infection (dotted line) and the HCV mono-infection group (solid line).

All patients infected with HIV were treated with combined ART according to currently accepted guidelines (2NRTI + PI or 2NRTI + NNRTI). Patients infected with HCV were treated for 48 week with pegylated IFN alfa-2a (180 μg/week) and ribavirin (900 to 1,200 mg/day, depending on body weight). HIV and HCV RNA levels were measured at six time points: before the start of therapy (baseline) and at 4, 12, 24, 36 and 48 weeks of antiretroviral therapy (Figure 1). HIV/HCV co-infected patients were more sensitive to HAART comparing with HIV mono-infected patients. However, after 48 weeks of HAART all patients had reached lower HIV-1 RNA level which could be considered as a great viral response to antiretroviral therapy (Figure 1A). HCV antiretroviral therapeutic situation indicated that HIV/HCV co-infected patients were slower to reach sustained virologic response (SVR) than HCV mono-infected patients (Figure 1B).

### Th1 and Th2 cytokine concentration between four groups at baseline

**Figure 2:**
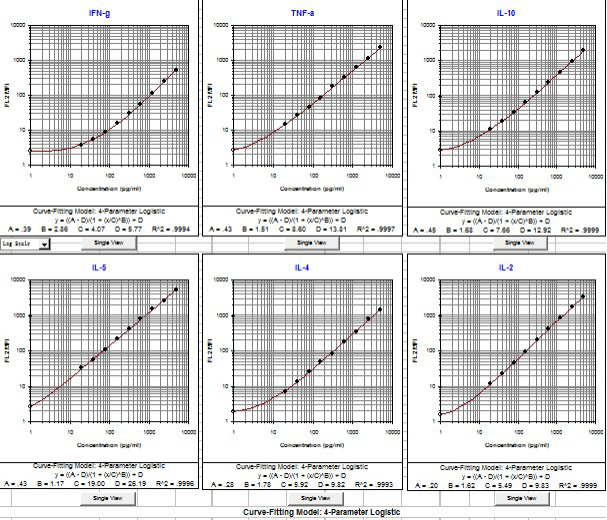
**Th1/Th2 Cytokine Standard curves generated by BD CBA Software**. Human Th1/Th2 Cytokine Standards were reconstituted in 2.0 ml of Assay Diluent; dilute the Standards primary liquid in a serial dilution rate as described: 1:2, 1:4, 1:8, 1:16, 1:32, 1:64, 1:128, and 1:256; prepare one tube containing Assay Diluent as negative control (0 pg/ml). 50 μl of the mixed Capture Beads, 50 μl of the Human Th1/Th2 PE Detection Reagent, and 50 μl of Cytokine Standard dilutions were mixed before acquiring by CellQuest software; standard curves were calculated using the BD CBA Software after acquiring, sample cytokine concentrations were calculated by standard curves.

**Figure 3:**
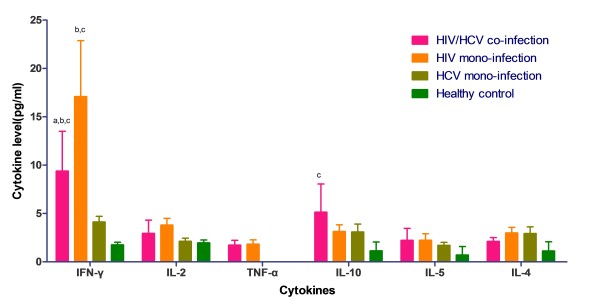
**Comparison of Th1 and Th2 cytokine levels between different groups at baseline**. Horizontal axis represents different kind of cytokines; vertical axis represents cytokine concentration (pg/ml) in plasma. Four coloured bar represents different study groups: HIV/HCV co-infection group (pink bars), HIV mono-infection group (orange bars), HCV mono-infection group (olive bars), and healthy control (green bars).

Six cytokines were detected using CBA Th1/Th2 cytokine kit, Th1/Th2 cytokine standard curves were shown in Figure 2. Cytokine concentration of plasma in patients was calculated according to the standard curves with 20 pg/ml ~ 5000 pg/ml quantifiable ranges. Results of six cytokines' concentration of plasma in four study groups were shown in Figure 3. IFN-γ concentration was significantly lower in HIV/HCV co-infection group comparing with HIV mono-infection group (*p *< 0.001), while higher comparing with both HCV mono-infection (*p *< 0.001) and healthy control group (*p *< 0.001). IL-10 concentration was highest in HIV/HCV co-infection group when compared with the other three groups, but it was only shown a statistical raise when compared with healthy control group (*p *< 0.01). In HIV/HCV co-infection group, IL-2 concentration was the highest without statistical difference (*p *> 0.05). Co-infection group and HIV mono-infection group revealed a slightly elevation of IL-5 concentration when compared with HCV mono-infection and healthy control group (*p *> 0.05), while the same level between HIV mono-infection. TNF-α concentration was detectable in the people who were infected with HIV. Co-infection group has lower IL-4 concentration compared with both HIV mono- and HCV mono-infection group, while higher than healthy control group.

**Figure 4:**
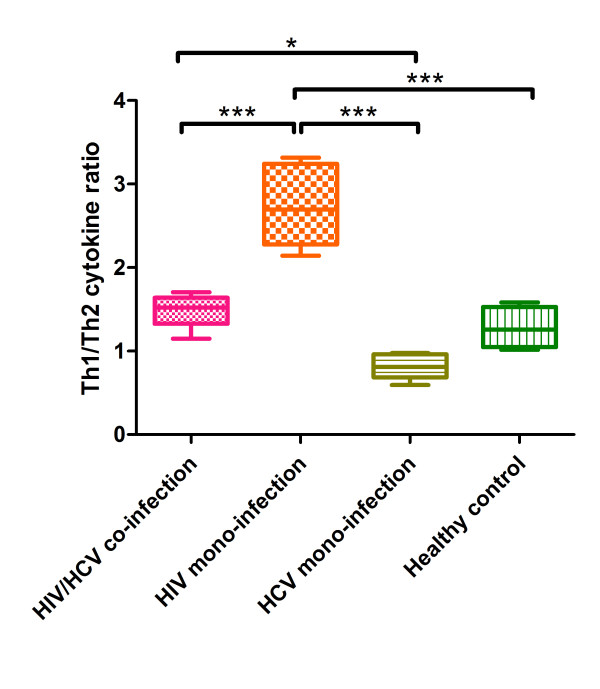
**Ratio of Th1 and Th2 cumulative concentration at baseline in different groups**. Horizontal axis represents four different study groups: HIV/HCV co-infection group (pink rectangle), HIV mono-infection group (orange rectangle), HCV mono-infection group (olive rectangle), and healthy control (green rectangle); vertical axis represents the ratio of Th1 and Th2 cytokine cumulative concentration at baseline. *: p < 0.05; ***: p < 0.001.

Furthermore, we estimated the ratio of Th1 and Th2 by calculating Th1/2 cytokines' concentration, thus to evaluate the change of Th1/Th2 expression balance. The ratio of Th1 and Th2 cytokine concentration was presented in Figure 4, illustrating the HIV mono-infection group had the highest ratio than the others (*p *< 0.001); the ratio of HIV/HCV co-infection group was significantly lower than HIV mono-infection group (*p *< 0.001) and higher than HCV mono-infection (*p *< 0.05). Comparing with healthy controls, infected with HIV could induce higher Th1 cytokine levels, however, infected with HCV could induce higher Th2 cytokine level. Considering these two opposite effects, we observed a coordinative result in the HIV/HCV co-infection group, which was higher than HCV mono-infection and lower than HIV mono-infection group.

### Dynamic changes of Th1 and Th2 cytokines during HAART

**Figure 5:**
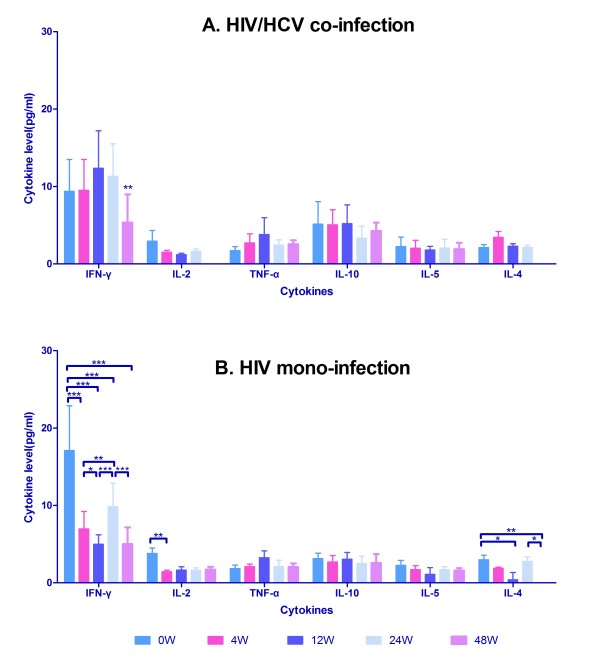
**Comparison of Th1 and Th2 cytokine concentration during HAART between HIV/HCV co-infection and HIV mono-infection group**. Horizontal axis represents six time points with different colour bars: before the start of therapy (0 W, Paris blue bars) and at 4 (pink bars), 12(blue bars), 24 (light blue bars), and 48 weeks (lavender bars) of HAART; vertical axis represents cytokine concentration (pg/ml) in plasma. (A) Dynamic change of Th1 and Th2 cytokine concentration during HAART in HIV/HCV co-infection group; (B) Dynamic change of Th1and Th2 cytokine concentration during HAART in HIV mono-infection group. *: p < 0.05; **: p < 0.01; ***: p < 0.001.

After initiating HAART, we estimated Th1 and Th2 cytokine levels of the HIV mono-infection and the HIV/HCV co-infection group at series time-points (baseline, 4 weeks, 12 weeks, 24 weeks and 48 weeks). Concentration of IFN-γ at 48 weeks after HAART in HIV/HCV co-infected patients indicated a significant decrease comparing with the other time points (*p *< 0.01) (Figure 5A), while the other cytokine levels presented no statistical differences. However, concentration of IFN-γ in HIV mono-infection group was more variable during HAART (Figure 5B). IFN-γ was secreted at the highest level at the baseline (*p *< 0.001), fell sharply at 4 weeks after HAART (*p *< 0.05), rebounded at 24 weeks (*p *< 0.001), and fell down again at 48 weeks (*p *< 0.001). Concentrations of IL-2 shown peak level at baseline, descended quickly at 4 weeks (*p *< 0.01), and ascended slightly as time-varying (*p *> 0.05). IL-4 was secreted at first (*p *< 0.05), descended progressively after HAART in 12 weeks (*p *< 0.05), rebounded abruptly at 24 weeks (*p *> 0.05), and fell off at 48 weeks (*p *< 0.05). TNF-α, IL-10 and IL-5 levels remained the same level of baseline as HAART was proceeding.

**Figure 6:**
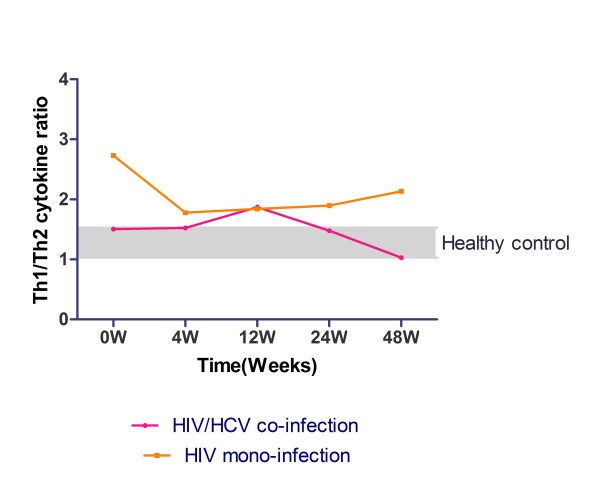
**Comparison of the dynamic change of Th1/Th2 cytokine ratio during HAART between HIV/HCV co-infection group and HIV mono-infection group**. Vertical axis represents the ratio of Th1 and Th2 cytokine cumulative concentration at baseline; two coloured lines represent different study group: HIV/HCV co-infection group (pink line), HIV mono-infection group (orange line). Gray zone was shown as the Th1/Th2 ratio range of healthy control.

The change of Th1/Th2 ratio of mono- and co-infection group was demonstrated in Figure 6, which indicated that co-infection group has a complete different pattern during HAART; Th1/Th2 ratio in co-infection group was stabilized at first then elevated at 12 weeks, and descended after 12 weeks; while in HIV mono-infection group Th1/Th2 ratio fell sharply in 4 weeks then kept stable after with a slightly ascending trend. After 48 weeks treatment of HAART, Th1/Th2 ratio of HIV/HCV co-infected patients stayed in the same level of healthy control group. However, HIV mono-infection group was still higher than the control group.

**Figure 7:**
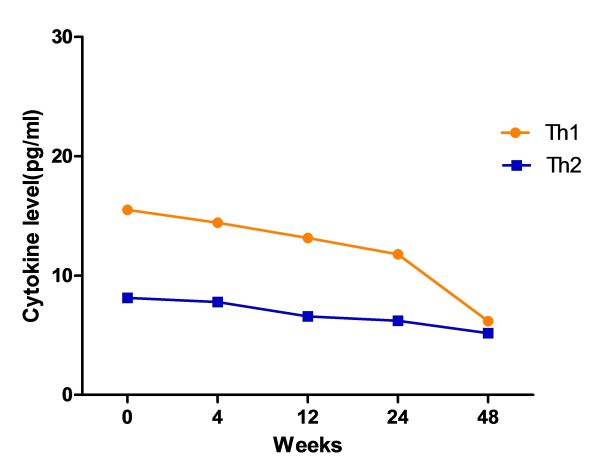
**Changes of Th1 and Th2 cytokine expression levels during HAART in both HIV mono-infection and HIV/HCV co-infection group**. Vertical axis represents cytokine concentration (pg/ml) in plasma. The orange line represents Th1 cytokine cumulative concentration (pg/ml); the blue line represents Th2 cytokine cumulative concentration (pg/ml).

Furthermore, we evaluated the changes of Th1 and Th2 cytokine levels of the entire HIV infected patients who had received HAART. There was an obviously time-varying descending pathway (Figure 7). Th1 cytokine levels seemed more sensitive to HAART than Th2.

### Relationships between Th1/Th2 cytokines, CD4+ T lymphocyte count and virus load

We analyzed the correlations between the main factors concerned, included CD4^+ ^T lymphocyte count, HIV viral load, concentration of Th1 and Th2 cytokines (IL-2, IL-4, IL-5, IL-10, TNF-α and IFN-γ) and Th1/Th2 ratio, the results were exhibited on Table 2. Statistic results indicated that HIV-1 viral load had strong positive correlation with most Th1 and Th2 cytokine levels, especially in IFN-γ (r = 0.537; *p *< 0.01), IL-2 (r = 0.592; *p *< 0.01), IL-10 (r = 0.381; *p *< 0.01) and IL-5 (r = 0.360; *p *< 0.01), while negative correlation with TNF-α (r = -0.405; *p *< 0.05). CD4^+ ^T lymphocyte count had significant negative correlation with HIV viral load (r = -0.519; *p *< 0.01), IL-2 (r = -0.335; *p *< 0.01) and IL-5 (r = -0.317; *p *< 0.01) concentration, and Th1/Th2 ratio (r = -0.236; *p *< 0.05). IFN-γ, IL-2, IL-10, Th1 cytokines levels and Th2 cytokines levels all had strong positive correlations with the cytokine concentration except TNF-α. Moreover, TNF-α was the most insensitive immune cytokine which did not correlated with the others except a negative correlation with HIV viral load (r = -0.405; *p *< 0.05). As the proceeding of HAART, HIV viral load was inhibited, CD4^+ ^T lymphocyte count aroused, TNF-α expression raised, the concentration of IFN-γ, IL-2, IL-5 and IL-10 progressively decreased. However, Th1 cytokine behaved more sensitive than Th2 cytokine under the effect of HAART. IFN-γ secretion had a strong and intense relationship with Th1 cytokine levels (r = 0.969; *p *< 0.01), which indicated that IFN-γ might play an important role in Th1/Th2 balance.

**Table 2:** Correlation between the concentration of Th1/Th2 cytokines, CD4^+ ^T lymphocyte count, virus load and Th1/Th2 cytokine ratio

Correlation Coefficient	CD4	VL	IFN-γ	TNF-α	IL-2	Th1 cytokines	IL-10	IL-5	IL-4	Th2 cytokines	Th1/Th2 ratio
CD4	1.000	-0.519^a^	-0.192	0.262	-0.335^a^	-0.112	-0.019	-0.317^a^	0.108	0.121	-0.236^b^

VL	-0.519^a^	1.000	0.537^a^	-0.405^b^	0.592^a^	0.534^a^	0.381^a^	0.360^a^	0.112	0.295^b^	0.383^a^

IFN-γ	-0.192	0.537^a^	1.000	-0.229	0.443^a^	0.969^a^	0.286^b^	0.315^a^	0.383^a^	0.498^a^	0.701^a^

TNF-α	0.262	-0.405^b^	-0.229	1.000	-0.257	-0.092	0.043	-0.115	0.157	0.117	-0.214

IL-2	-0.335^a^	0.592^a^	0.443^a^	-0.257	1.000	0.407^a^	0.510^a^	0.430^a^	0.298^b^	0.334^a^	0.287^b^

Th1 cytokines	-0.112	0.534^a^	0.969^a^	-0.092	0.407^a^	1.000	0.270^b^	0.278^b^	0.329^b^	0.486^a^	0.775^a^

IL-10	-0.019	0.381^a^	0.286^b^	0.043	0.510^a^	0.270^b^	1.000	0.362^a^	0.290^b^	0.690^a^	-0.155

IL-5	-0.317^a^	0.360^a^	0.315^a^	-0.115	0.430^a^	0.278^b^	0.362^a^	1.000	0.233	0.433^a^	0.139

IL-4	0.108	0.112	0.383^a^	0.157	0.298^b^	0.329^b^	0.290^b^	0.233	1.000	0.668^a^	-0.009

Th2 cytokines	0.121	0.295^b^	0.498^a^	0.117	0.334^a^	0.486^a^	0.690^a^	0.433^a^	0.668^a^	1.000	-0.104

Each data represents the correlation coefficient betwwen horizontal and vertical effectors which are overlapped.

a. Correlation is significant at the 0.01 level (2-tailed)

b. Correlation is significant at the 0.05 level (2-tailed)

## Discussion

Although HIV co-infection clearly accelerates progression of HCV-related fibrosis and liver disease [35-38], the role of HCV in HIV disease progression remains controversial. HIV and HCV are intracellular parasites, therefore T-cell responses are crucial for antiviral defense and cytotoxic T lymphocyte (CTL) play a principal task by destructing infected cells [39,40]. Moreover, viral gene expression can be suppressed by the antiviral cytokines. Thus, both of the quantity and quality of T cells are essential on disease pathology [41]. Cytokines derived from CD4+ T cells play a prominent role in viral infection, fostering elimination of intracellular pathogen and promoting host viral responses including CTL generation and natural killer cell activation (Th1), or driving humoral immune responses and inhibit the development of Th1 responses (Th2) [42]. Therefore, it seems to be a delicate balance between the beneficial and detrimental effects of HCV co-infection on the HIV related disease progression and therapeutic results which require further study.

To investigate the imbalance of Th1 and Th2 cytokines in the peripheral blood of patients with HIV and/or HCV infection, we evaluated the cytokine levels by using flow cytometry in plasma, as well as the dynamic changes of cytokine concentrations during HAART. We found that HIV/HCV co-infected group had statistically higher CD4^+ ^T lymphocyte count, lower HIV viral load, and older mean age than HIV mono-infected patients. This fact may be an indirect evidence to support that co-infection with HCV has no effect on the progression of HIV to AIDS. We also found a higher detection level of HCV RNA in the HCV/HIV co-infected group compared to the HCV mono-infected group; these findings are consistent with other studies [43]. At the baseline level, co-infected with HCV can decrease the cytokine levels produced by both Th1 and Th2 cells comparing with HIV mono-infection except IL-10; HIV mono-infection had the highest Th1 immune responses by secreting IFN-γ compared with other groups; Th2 cytokine levels were lower than Th1, demonstrated a profile dominated by Th1 cytokines before HAART. HIV or HCV mono-infection with higher Th1 cytokine expressions may account for a milder course of liver disease in HCV mono-infection [33,44]; Th1 to Th2 switch led to the contraction of AIDS [45]. Therefore, we suggested that co-infection with HIV didn't worsen liver diseases by the performance of cytokine levels.

HIV infection specifically depressed CD4+ T cell and kept viral replication at high levels, which ultimately led to AIDS syndromes. On the other hand, many studies found that infected with HIV could cause cytokine expression of Th1 to Th2 during progresses of AIDS. By analyzing the former factors related with diseases progression. We found that HIV/HCV co-infection group had lower Th1/Th2 ratio, the CD4^+ ^T lymphocyte count and HIV viral load was higher than HIV mono-infected group. Therefore, we reached a conclusion that there's no sufficient corroboration for that co-infected with HCV has negative effect on HIV related diseases. As we mentioned before, the influence of HCV on HIV infected patients was probably in the functional disorder of the T cells, which can not be determined by the quantity of cytokine levels. Infected with different viruses could lead to different characteristic performances of Th1/Th2 imbalance. Contrasting with the healthy control, the cytokine levels were dominated by Th1 cytokines in HIV mono-infection group, while in HCV mono-infection the advantage of Th1 was replaced by Th2 cytokines, thus for co-infection group Th1/Th2 balance was medium between the two mono-infections. These findings were similar to the study of P. Price et al. [34], which indicated that compared with single infected patients, the T helper response of co-infected with HIV and HCV was markedly reduced. Thus, co-infected with HCV could cause a lower Th1/Th2 ratio by decreasing Th1 cytokine expressions, especially in the IFN-γ level. HIV/HCV co-infection and HIV mono-infection group presented differently after HAART. As HAART successfully inhibited HIV replication and elevated CD4^+ ^T lymphocytes, the reactivity of Th1 and Th2 cells was changed evidently from baseline. Co-infected with HCV seemed to play a role in deregulating both Th1 and Th2 cytokine expressions. Th1/Th2 balances in different study groups during HAART were also performing in two different ways, which implied co-infected with HCV affected mostly on Th1 cytokine expressions leading to a progressive decrease. The strong and positive correlation between HIV viral load and Th1 and Th2 cytokine levels indicated that HIV was an essential influencing factor on the reactivity of Th1 and Th2 cells. Therefore, Th1 and Th2 cytokine levels were reduced as HAART proceeding. As there's no significant correlation between CD4^+ ^T lymphocyte count and Th1 and Th2 cytokine levels, we proposed the possible reason for increasing cytokine expression was the HIV itself. It was the stimulation of the HIV protein on T cells that caused the high cytokine expressions, which needed further study to reveal the molecular mechanism of this effect.

## Conclusions

In summary, this study investigated the differences of Th1 and Th2 cytokine levels between HIV/HCV co-infection and HIV or HCV mono-infection group as well as the change of Th1/Th2 balance during HAART. There was no significant evidence that co-infected with HCV had any negative effects on HIV related diseases. However, co-infected with HCV can decrease Th1/Th2 ratio by affecting Th1 cytokine expression. With initiating of HAART, Th1 and Th2 cytokine levels were progressively reduced. HIV was the main stimulation factor of T cells in co-infection. Additional studies are needed to illustrate the mechanism behind reduced HCV-specific T helper responses, as well as the clinical implications of reduced cytokine responses in co-infected patients.

## Competing interests

The authors declare that they have no competing interests.

## Authors' contributions

YZ recruited the subjects and collected the samples. KZ, DH performed the CD4 cell count and viral load tests. YL, YZ carried out the test of Th1 and Th2 cytokines. WK, YL, YZ, DH carried out the data analysis and drafted the manuscript. WK, YS conceived of the study, and participated in its design and coordination. All authors read and approved the final manuscript.

## Pre-publication history

The pre-publication history for this paper can be accessed here:

http://www.biomedcentral.com/1471-2334/12/102/prepub
